# M/EEG Dynamics Underlying Reserve, Resilience, and Maintenance in Aging: A Review

**DOI:** 10.3389/fpsyg.2022.861973

**Published:** 2022-05-25

**Authors:** Gwendolyn Jauny, Francis Eustache, Thomas Thierry Hinault

**Affiliations:** Normandie Univ, UNICAEN, PSL Université Paris, EPHE, INSERM, U1077, CHU de Caen, Centre Cyceron, Caen, France

**Keywords:** aging, cognition, M/EEG, dementia, connectivity

## Abstract

Cognitive reserve and resilience refer to the set of processes allowing the preservation of cognitive performance in the presence of structural and functional brain changes. Investigations of these concepts have provided unique insights into the heterogeneity of cognitive and brain changes associated with aging. Previous work mainly relied on methods benefiting from a high spatial precision but a low temporal resolution, and thus the temporal brain dynamics underlying these concepts remains poorly known. Moreover, while spontaneous fluctuations of neural activity have long been considered as noise, recent work highlights its critical contribution to brain functions. In this study, we synthesized the current state of knowledge from magnetoencephalography (MEG) and electroencephalography (EEG) studies that investigated the contribution of maintenance of neural synchrony, and variability of brain dynamics, to cognitive changes associated with healthy aging and the progression of neurodegenerative disease (such as Alzheimer's disease). The reviewed findings highlight that compensations could be associated with increased synchrony of higher (>10 Hz) frequency bands. Maintenance of young-like synchrony patterns was also observed in healthy older individuals. Both maintenance and compensation appear to be highly related to preserved structural integrity (brain reserve). However, increased synchrony was also found to be deleterious in some cases and reflects neurodegenerative processes. These results provide major elements on the stability or variability of functional networks as well as maintenance of neural synchrony over time, and their association with individual cognitive changes with aging. These findings could provide new and interesting considerations about cognitive reserve, maintenance, and resilience of brain functions and cognition.

## Introduction

Understanding the evolution of cognition as we age is crucial not only because it is intimately related to everyone's subjective experience, but to help describe and better understand cognition itself. Cognitive aging is a highly variable phenomenon, characterized by a decline in cognitive abilities that might lead to higher risk of pathological aging in some individuals, whereas others are able to remain efficient in most everyday tasks until an advanced age (Reuter-Lorenz and Park, 2014). Research on the variability of age-related changes across individuals aims at answering several questions, such as the factors associated with preserved cognitive functioning with age (the “*why*” question), or what differs between individuals with preserved vs. declined cognitive performance (the “*how*” question). While lifestyle activities associated with reduced risk of cognitive decline and pathological aging have been extensively investigated and specified (see Livingston et al., 2020, for a recent review), a mechanistic understanding of the neural bases underlying individual differences with advancing age remains to be further developed. Achieving this goal requires the use of highly sensitive methods, and the investigation on brain activity with high spatial and temporal resolutions.

The concept of cognitive reserve has originally been proposed to improve our understanding of mechanisms underlying sustained cognitive performance in spite of age-related and pathology-related brain changes (Stern, 2002). Over the last two decades, this concept has undergone several changes and developments (see Cabeza et al., 2018; Stern et al., 2020; Pascual-Leone and Bartres-Faz, 2021). The current concepts of maintenance, resistance, and resilience have been proposed to specify the heterogeneity brain and cognitive changes in face of aging and neurodegenerative disease. During healthy aging, *maintenance* (Nyberg et al., 2012) refers to the relative absence of brain changes with advancing age, whereas *reserve* (Stern, 2002) encompasses the compensatory processes allowing the preservation of cognitive performance in the presence of structural and functional brain changes. The concept of reserve involves two complementary aspects, namely, brain reserve (i.e., individual differences in brain size, number of synapses, etc.) and cognitive reserve (i.e., individual differences in the cognitive processes and brain networks recruited to perform a given task). With the progression of neurodegenerative disorders, *resistance* (Arenaza-Urquijo and Vemuri, 2018) defines the protection of brain reserve against alterations and the prevention of brain lesions and damages before their occurrence. Finally, *resilience* (Pascual-Leone and Bartres-Faz, 2021) concerns the processes involved in the compensation of changes occurring with the development of neurodegenerative disorders, explaining various degrees of symptomatology in the face of an equal pathological load. Some degree of overlap may exist between these concepts. As an example, compensatory adjustments (e.g., increased frontal recruitment) might be implemented in the presence of both age- and pathology-related changes, yet qualitative differences may also be observed between the adjustments implemented in response to healthy aging and pathology. A recent review of Pascual-Leone and Bartres-Faz (2021) specified these concepts and emphasized the need for individually based markers that could predict an individual's risk for disability and cognitive decline.

Neuroimaging studies have been central in the emergence and evolution of the concepts of reserve, maintenance, and resilience. Several decades of work aimed at investigating structural (i.e., gray matter, white matter) and functional (i.e., local activity, association between the activities of distinct brain regions) changes with age and pathology (e.g., Damoiseaux, 2017; Spreng and Turner, 2019). Recent work revealed that these dimensions are closely related, with large-scale brain functional network relying on a structural architecture of white matter fibers (e.g., Hinault et al., 2019; Suárez et al., 2020). Indeed, preserved brain integrity has been associated with preserved cognitive functioning. This work also identified compensatory adjustments in older adults (i.e., larger activations, bilateral recruitment) associated with similar cognitive performance to that of younger individuals (e.g., Cabeza et al., 2018). These studies mainly relied on high spatial resolution methods such as functional magnetic resonance imaging (fMRI), characterized by excellent spatial accuracy but low temporal resolution (in the order of seconds). Therefore, the temporal dynamics of brain networks and the influence of age and pathology on these dynamics remain poorly known. Investigating these dynamics with high temporal resolution methods (in the order of milliseconds) such as magnetoencephalography (MEG) or electroencephalography (EEG) is important because higher cognitive functions such as memory and cognitive control that are most affected by age and pathology involve multiple cognitive processes in rapid successions and short durations (e.g., Courtney and Hinault, 2021). Even when compensation may mask these changes in clinical or neuropsychological assessment, specifying dynamic network connectivity may reveal alterations in the stability of communications between brain regions or delays in the fast succession of connectivity patterns, providing a better understanding of the concepts of reserve, maintenance, resilience, and resistance.

The brain generates its own temporal structure, which is critical to the ways in which signals are routed, combined, and coordinated (e.g., Bao et al., 2015; Voytek and Knight, 2015). Brain rhythms present the particularity of being observed across species (e.g., Buzsáki, 2019), which suggest a major evolutionary advantage of rhythmic communications (e.g., Miller et al., 2018). Brain rhythms are distinguished in different frequency bands [delta (1–4 Hz), theta (4–8 Hz), alpha (8–12 Hz), beta (12–30 Hz), gamma (30–45 Hz)]. MEG and EEG methods enable the investigation of these rhythms and dynamic connectivity patterns in humans (e.g., Baillet, 2017). Given the critical involvement of coordinated brain rhythms in higher-order cognitive processing, age-related changes in the dynamic of brain communications may act as a marker for cognitive decline in older adults. Such complex interplay across rhythms is highly sensitive to structural changes and the progression of neurodegenerative disorders (e.g., Gaubert et al., 2019; Courtney and Hinault, 2021) as even small changes of dynamic synchrony can lead to cognitive decline with aging (e.g., Hinault et al., 2020, 2021; Kumral et al., 2020).

In this study, we reviewed EEG and MEG studies in healthy older individuals and in patients to identify elements that shed new light on cognitive aging and pathology effects. We focused on three main patterns, namely, (1) compensatory or resilience adjustments when brain dynamics are altered, (2) maintenance of these dynamics in the face of aging or pathology, and (3) non-effective or maladaptive changes related to normal aging or pathology. These concepts have been extensively investigated with fMRI; we thus aimed at highlighting the complementary contribution of M/EEG findings. M/EEG can indeed detect between-group differences in the initiation, maintenance, or interruption of brain communication at the sub-second level, which is therefore highly sensitive to early disruptions associated with age and pathology. In this review, we first presented studies contributing to these concepts in healthy aging before presenting work on pathological aging. Given the work conducted in pathological aging, we focused on Alzheimer's disease (AD) and its preclinical stage the mild cognitive impairment (MCI). We concluded with recommendations and future directions that could guide and stimulate future studies. Such endeavor could lead to new cognitive stimulations strategies targeting brain rhythms and reduce the cognitive decline commonly associated with age.

## Normal Aging

**Figure 1:**
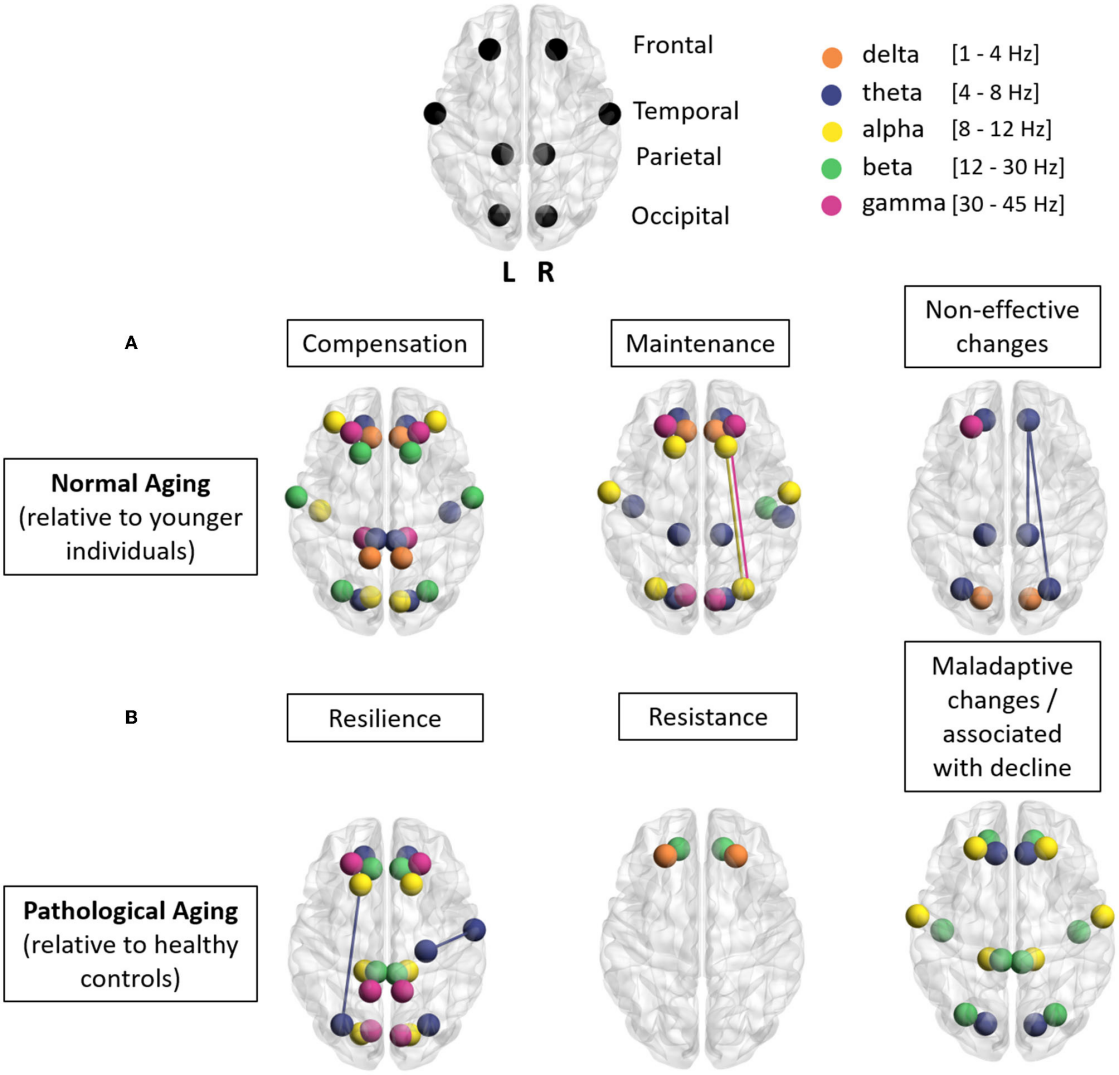
Illustration of the main results of this study: each point represents a brain region and each color a frequency band (orange, delta; blue, theta; yellow, alpha; green, beta; pink, gamma). **(A)** Normal aging. This row of three brains summarizes the main results found in this review (change in synchrony in different regions for different frequency bands relative to younger individuals) for the notions of compensation, maintenance, and non-effective changes in normal aging. **(B)** Pathological aging. This row of three brains summarizes the main results found in this review (change in synchrony in different regions for different frequency bands, relative to healthy controls) for the notions of resilience, resistance, and maladaptive changes in pathological aging. Using BrainNet-viewer tool (Xia et al., 2013).

Nineteen M/EEG studies, including younger (20–30 years) and older (60–85 years) healthy participants, have been selected, investigating cognitive performance and neural synchrony data (see Table 1). In this study, we described three of the most commonly used techniques. First, the phase-locking value (PLV; Lachaux et al., 1999) measures whether the oscillatory phase of the activity of a group of neurons, in a certain frequency band, can transiently synchronize with those of another group of neurons. This transient phase locking has been associated with communications between neural groups (Fries, 2015). A second technique similar to this is the phase lag index (PLI; Stam et al., 2007), which measures the asymmetry of the distribution of phase differences between two signals. Finally, coherence (Nunez et al., 1997) measures the association of signals' amplitudes across brain regions. These measures and provides information about neural synchrony and the presence of functional connectivity between brain regions. The main results studied in this section are illustrated in Figure 1A.

**Table 1:** Summary of the results.

**References**	**Measure**	**Method**	**Task**	**Participants (age)**	***N***	**Results**
**Healthy aging**						
**Compensation**						
Ariza et al. (2015)	MEG	PLI	Working memory	Young healthy adults (21.88) Older healthy adults (64.45)	9 11	The global increase in alpha synchrony is positively correlated with the maintenance of cognitive performance in the elderly group
Aktürk et al. (2020)	EEG	Coherence	Facial expressions recognition task	Young healthy adults (24) Older healthy adults (62.07)	15 15	Increased delta, theta, and alpha synchrony in frontal position in healthy elderly would allow maintenance of cognitive performance compared to the young
Hong et al. (2016)	EEG	Phase synchronization analysis	Go/NoGo	Young healthy adults (21.4) Older healthy adults (61)	23 18	Increased delta and theta phase synchronization in the fronto-central and parieto-central areas is associated with the maintenance of cognitive performance for the elderly group
Phillips and Takeda (2010)	EEG	PLV	Visual search	Older healthy adults (68)	14	Increased gamma synchronization in the fronto-parietal position is positively correlated with cognitive performance in the elderly group
Rosjat et al. (2018)	EEG	PLV	Finger tap	Young healthy adults (22–35) Older healthy adults (60–78)	21 31	Maintenance of the increase in delta synchronization in the elderly (similar to the young group) could enable the maintenance of cognitive performance in this same group compared to the young group
Rosjat et al. (2021)	EEG	PLV	Finger tap	Young healthy adults (22–35) Older healthy adults (60–78)	21 31	Maintenance of the increase in delta and theta synchronization in the elderly (similar to that of the younger group) would allow the maintenance of cognitive performance
**Maintenance**						
Coquelet et al. (2017)	MEG	Power envelope correlation	Resting state	Young healthy adults (23.6) Older healthy adults (68.8)	25 25	Maintenance of synchrony in beta band in elderly at a similar level than the young group is positively correlated with cognitive performance
Hinault et al. (2020)	EEG	PLV	Working memory	Young healthy adults (23.2) Older healthy adults (71.0)	40 40	Older with preserved integrity of white matters fibers show preserved functional synchrony in alpha and gamma band and no difference in cognitive performance with younger participants
Ho et al. (2012)	EEG	Phase locking measures and coherence	Attention task	Young healthy adults (23.7) Older healthy adults (70.1)	15 15	Decreased theta and alpha synchronization in the parietal region are positively correlated with cognitive performance in the elderly group (No difference in cognitive performance between groups)
Liu et al. (2017)	EEG	PLI	Finger tap	Young healthy adults (22–35) Older healthy adults (60–78)	18 24	Maintenance of delta and theta synchronization of central regions in the elderly group associated with poorer cognitive performance compared to the young group
López et al. (2014b)	MEG	PLV and PLI	Sternberg task	Older healthy adults high cognitive reserve (67.3) Older healthy adults low cognitive reserve (69.7)	9 12	The low reserve group shows an increase in theta (fronto-parietooccipital), alpha (fronto-temporo-occipital) and beta (parieto-fronto-temporo-occipital) connectivity would allow maintenance of cognitive performance compared to the high reserve group
Rondina et al. (2019)	MEG	Phase synchronization analysis	Spatial memory	Young healthy adults (24.8) Older healthy adults (65.9)	16 16	Increased theta synchrony in occipital regions and decreased theta synchrony in fronto-temporo-parietal regions in the elderly in a manner similar to the young group allows maintenance of cognitive performance for the older group
**Non-effective changes**						
Hinault et al. (2021)	M/EEG	PLV	Working memory	Young healthy adults (23) Older healthy adults (71)	40 40	Decreased structural integrity in the elderly group alters the stability of communications (alpha and gamma frequency bands) between brain regions compared to the young group
Jauny et al., 2022	MEG	PLV and TE	Resting state	Young healthy adults (26.5) Older healthy adults (64.5)	46 46	Increased variability of delta synchrony in the DMN network and reversal of information transfer in the anterior to posterior direction of functional connectivity with age correlates with decreased cognitive performance
Li and Zhao (2015)	EEG	PLV	Visual search	Young healthy adults (23.9) Older healthy adults (63.1)	13 13	Increased theta and alpha centro-frontal synchrony and decreased beta centro-parietal synchrony in the older group could explain the decreased cognitive performance compared to the younger group
Paul et al. (2011)	EEG	Phase synchrony	Maze test	Young healthy adults (24.6) Older healthy adults (58)	160 100	Increased gamma2 synchrony in left frontal areas correlated with decreased cognitive performance for the older group
Sahoo et al. (2020)	MEG	Coherence	Resting state	Young healthy adults (18–35) Older healthy adults (66–88)	126 216	The decrease in alpha synchrony correlates with the decrease in cognitive performance on the VSTM test
Tóth et al. (2014)	EEG	PLI	Working memory	Young healthy adults (21.1) Older healthy adults (65.8)	20 16	Decreased theta synchronization between frontal and posterior regions could explain the decreased cognitive performance in the older group
Wessel et al. (2016)	EEG	PLV	Cognitive control	Older healthy adults (62) Older adults with focal frontostriatal lesion of withe matter (62.42)	12 12	The lesion group shows a decrease of beta synchrony
**Pathological aging**						
**Resilience**						
Bajo et al. (2010)	MEG	Synchronization Likelihood	Memory task	Older healthy adults (71.6) MCI patients (74.8)	19 22	Increased alpha, beta and gamma synchronization in MCI participants is associated to the preservation of cognitive performance compared to the healthy elderly group
Gaubert et al. (2019)	EEG	PLV	Resting state	Older healthy adults (75.62) preclinical AD patients (76.88)	175 25	Increased alpha synchrony in preclinical ADs with low amyloid levels would allow preserve of cognitive performance at a level similar to healthy elderly. Amyloid deposits could have a negative impact on the extracellular environment, preventing compensatory adjustments.
Rondina et al. (2019)	MEG	Phase synchronization analysis	Spatial memory	Young healthy adults (24.8) Older healthy adults (65.9)	16 16	Increased theta synchrony in occipital regions and decreased theta synchrony in fronto-temporo-parietal regions in the elderly in a manner similar to the young group allows maintenance of cognitive performance for the older group
López et al. (2014a)	MEG	PLV	Resting state and working memory	Older healthy adults (71.8) MCI patients (72.5)	32 38	An overall increase in theta synchrony was reported in the right fronto-occipital and parieto-temporal regions for the MCI group, as well as an increase in delta synchrony in the interhemispheric frontal regions. All this would allow the MCI group to have similar cognitive performance to the healthy elderly group
**Resistance**						
Knyazeva et al. (2013)	EEG	S estimator	Resting state	Older healthy adults (67.6) AD patients (68.7)	15 15	Increased synchrony in individuals with MCI was initially associated with preservation of brain and cognitive functioning, but may also be a sign of disease progression
López et al. (2014a)	MEG	PLV	Resting state and working memory	Older healthy adults (71.8) MCI patients (72.5)	32 38	An overall increase in theta synchrony was reported in the right fronto-occipital and parieto-temporal regions for the MCI group, as well as an increase in delta synchrony in the interhemispheric frontal regions. All this would allow the MCI group to have similar cognitive performance to the healthy elderly group
**Maladaptive changes**						
Caravaglios et al. (2018)	EEG	coherence	Resting state and omitted tone task	Older healthy adults (69.6) aMCI patients (68.1)	15 15	Increase in beta synchrony in aMCI patients correlated with poorer performance on various neuropsychological tests
Garn et al. (2015)	qEEG	Coherence	Resting state	AD patients (76) probable AD patients (75)	118 79	qEEG synchrony markers could predict AD severity
Houmani et al. (2018)	EEG	Bump model	Resting state	SCD participants (68.9) AD patients (81.6)	22 49	Discrimination of SCD and AD groups based on synchrony measures
Li et al. (2019)	EEG	PLI	Digit verbal span task	Older healthy adults (62.75) mild AD patients (72.5)	8 6	The decrease of connectivity in the alpha and beta frequency bands in frontal and parieto-temporal areas is correlated with impaired cognitive performance
Teipel et al. (2009)	EEG	Coherence	Resting state	Older healthy adults (67.0) aMCI patients (73.6)	20 16	The decrease of white matter integrity causes a decrease in alpha synchrony for aMCI participants

*EEG, electroencephalography; MEG, magnetoencephalography; PLI, phase lag index; PLV, phase-locking value; TE, transfer entropy*.

### Compensation

In older individuals with similar levels of cognitive performance as younger individuals, previous MRI studies have shown that older individuals frequently engage additional brain regions, as described in the Compensation Related Utilization of Neural Circuit Hypothesis (Reuter-Lorenz and Cappell, 2008) model. This increased recruitment of brain regions has been associated with cognitive performance and is referred to as compensation (Cabeza et al., 2018). Angel et al. (2010, 2011) investigated this phenomenon in EEG using event-related potential (ERP). They revealed larger bilateral activity in older individuals with higher cognitive performance. M/EEG studies critically identified that an increased synchronization of frontoparietal regions has been observed in older individuals showing preservation of cognitive functioning. Ariza et al. (2015) asked healthy young and elderly participants to perform an interference-based working memory task in which participants were asked to memorize a face/sound pair (a face was displayed and associated with a semantic attribute). The pair was followed by an interference phase during which a known face with a question about that person was presented to them. Finally, a recognition phase in which participants were asked to tell whether the presented pairs were the same as those presented in the encoding phase. This study reported a global increase in alpha synchrony (measured by PLV) in older individuals compared to younger individuals, whereas both groups showed similar cognitive performance. This increased alpha synchrony has only been observed during the interference period and has been interpreted as a compensatory mechanism in older individuals.

Using an inhibitory task, Hong et al. (2016) showed that an increase in phase synchrony of the delta and theta frequency bands localized at the frontocentral and parietocentral areas reflected a compensatory phenomenon in healthy older individuals (see also Phillips and Takeda, 2010, for similar findings in the gamma frequency band). Also, a posterior-anterior synchrony switch (increase in delta synchrony in anterior vs. posterior position) was also found to be compensatory for a facial emotion recognition task (Aktürk et al., 2020). These compensatory phenomena are found not only in cognitive tasks but also in motor tasks. For example, Rosjat et al. (2021) showed increases in delta and theta synchrony (measured by PLV) in healthy elderly in association with sustained motor performance at the same level as in young people (see also Rosjat et al., 2018).

### Maintenance

Increased brain activity is not systematic, and it is important to note that some individuals do not need to implement these compensations, possibly because of higher cognitive reserve levels (Stern, 2009). López et al. (2014b), in a study comparing the global connectivity (with PLV and PLI) of older participants with higher or lower cognitive reserve, showed compensatory phenomena from the latter group only. Indeed, while cognitive performance was similar between low and high reserve participants, individuals with lower reserve showed an increase in theta, alpha, and beta synchrony in frontal, temporal, and occipital regions. This suggests that some individuals show a more efficient network functioning and therefore do not need to engage a more extended brain network. Relatedly, Ho et al. (2012) showed that lower theta and alpha synchrony levels (measured with coherence and phase-locking measures) in the parietal region were associated with similar performance than younger individuals.

Maintenance thus refers to the preservation of similar brain activity levels than younger individuals, allowing cognitive abilities to be relatively preserved with advancing age (Nyberg et al., 2012). MRI work showed, for example, that older individuals with little changes in functional activity during encoding, relative to younger adults, can sometimes perform better than older individuals with larger activation changes (Düzel et al., 2011). Studies on this phenomenon are, to our knowledge, rare. Rondina et al. (2019) investigated the preservation of brain dynamics with aging, using a spatial memory task where participants were asked to remember the spatial location of three objects seen separately. In the test phase, participants were then asked to determine whether the location of the objects was the same or different than in the memory phase. They revealed that older individuals showed a similar pattern of brain activations than young participants, with an increase in theta band oscillatory activity in occipital regions and a decrease in frontotemporal regions. The maintenance of this brain activity pattern was associated with similar cognitive performance between age groups. Also, in a visual attention task, young and older individuals showed a similar pattern of increased lower gamma frequency activity in the frontal region with increasing task difficulty (Phillips and Takeda, 2010). Moreover, Coquelet et al. (2017) investigated MEG phase synchrony at rest and reported maintenance of the electrophysiological connectome with age. Coquelet and colleagues observed similar patterns between age groups in the alpha and beta frequency bands. The maintenance of synchrony in the beta band was positively correlated with the maintenance of cognitive performance in older individuals.

This functional maintenance seems to critically depend on the maintenance of the underlying structures of the brain networks. Hinault et al. (2020) investigated the structure-function relationship between diffusion tensor imaging (DTI) and EEG data of neuronal synchrony (PLV) of young and old healthy participants. The authors used an arithmetic task, in which participants had to maintain, or update, arithmetic operations (addition, multiplication) in working memory to determine whether the proposed equation results (e.g., 8 × 4 = 36) were correct or incorrect. Preserved integrity of white matter fibers at a similar level to younger participants, in some older individuals, was associated with preserved functional synchrony in the alpha and gamma frequency bands, and similar cognitive performances between age groups. In particular, the inferior fronto-occipital tract was associated to functional coupling between the inferior frontal gyrus and the occipital lobe. These results also illustrate the strong association with the preservation of brain reserve, which would enable such maintenance (Stern, 2009).

Thus, the maintenance of functional synchrony with age (at rest and during task completion) is associated with the preservation of cognitive functions in healthy elderly individuals. This maintenance has mainly been observed between frontal and parietal regions. However, the preservation of this functional synchrony at a similar level to younger individuals is frequently associated with an increased synchrony in other brain regions. Liu et al. (2017) showed that a maintenance of delta and theta synchrony of central regions (measured with the PLI), in the absence of increased activity in other regions, was associated with decreased motor and cognitive performance. This suggests that maintenance is strongly linked to the presence of functional compensations. However, such increased brain activity could also be deleterious, reflecting network alteration and the presence of excitotoxic or neurodegenerative phenomena (Hillary and Grafman, 2017).

### Non-Effective Changes

With advancing age, changes in brain function can be compensatory (as discussed above), but may also turn out to be ineffective. In an EEG study (Tóth et al., 2014), young and old healthy participants had to perform a working memory task consisting of remembering colored squares at certain locations. During the test phase, these squares remained at the same locations but could change in color. Participants had to determine whether squares were identical to those displayed during the encoding phase or not. Synchrony was determined with the PLI measure, and a specific decrease in theta synchrony between frontal and posterior sites was observed in healthy older adults compared to younger individuals (see also Li and Zhao, 2015). Importantly, this theta synchrony decrease has been associated with lower cognitive performance. Also, increased high gamma synchrony in the left frontal region during a maze task (visual planning task) has been shown to be negatively associated with performance (Paul et al., 2011).

In one of our studies (Jauny et al., 2022), we showed that maladaptive changes can be identified in resting-state activity. This study involved the Cam-CAN cohort (e.g., Shafto et al., 2014; Taylor et al., 2017) and changes in synchrony (with the PLV measure), as well as directed connectivity (with the entropy transfer measure, which provides a directed connectivity measure), were investigated. We observed a greater variability in brain networks' synchrony over time relative to younger individuals, mainly for the default mode network (this network is mainly activated when no task is proposed from the participant and plays a role in continuous environmental monitoring; Uddin et al., 2019) in the delta frequency band. Moreover, we showed that older individuals showed a revered dominant direction in information transfer, relative to younger individuals, with a significant increase in the anterior to posterior direction of functional connectivity. These changes were correlated with a decline in cognitive performance on tests measuring working memory, attention, and logical reasoning (also Sahoo et al., 2020). This reversal of the dominant information transfer direction could represent a failed compensation attempt. The increases in brain synchrony studied could also reflect excitotoxic processes (Hillary and Grafman, 2017; Mai et al., 2019), with increased activity preceding neuronal loss. Indeed, older individuals showing these hyperconnectivity phenomena had decreased cognitive performance.

As we have seen in these different sections, changes in synchrony vary over time and with task difficulty, and increased synchrony is not always beneficial. These changes appear to be related to changes in the level of white matter fibers integrity (Hinault et al., 2020, 2021). Indeed, the presence of white matter lesions has been associated with decreased brain synchrony (Wessel et al., 2016). In association with the influence of potentially preclinical excitotoxic processes, structural and functional changes could reflect the progression of neurodegenerative disorders.

## Pathological Aging

In this section, we considered M/EEG study involving patients with AD and its prodromal stage called mild cognitive impairment. AD is the most common age-related pathology and is characterized by memory and cognitive control impairment (involving inhibition, planification, and mental flexibility; Baudic et al., 2006). The investigation of early AD stages such as the MCI stage is of significant interest because some individuals will later develop AD whereas others will remain stable or develop other pathologies such as frontotemporal or vascular dementia (e.g., Perez-Gonzalez et al., 2014; Jongsiriyanyong and Limpawattana, 2018). The reviewed studies were selected based on the same criteria as in the previous section on normal aging (see Table 1). The main results studied in this section are illustrated in Figure 1B.

### Resilience

In line with the reserve concept developed in healthy older individuals, resilience refers to the processes involved in coping with the development of neurodegenerative disorders and the associated brain changes (Pascual-Leone and Bartres-Faz, 2021). Indeed, resilience allows, like reserve, the preservation of cognitive functions in spite of brain changes. Few studies have documented this phenomenon. Gaubert et al. (2019) compared resting-state EEG data (phase synchrony calculated using PLV and PLI) from patients with preclinical AD (at two AD progression stages) and control participants. They observed that the degree of neurodegeneration (measured by the level of amyloid beta load) impacted functional connectivity. Higher alpha functional connectivity in parieto-occipital sites was observed when amyloid load levels were low. This increase in alpha connectivity was interpreted as reflecting a compensatory phenomenon as patients showed normal cognitive performance (measured by the Free and Cued Selective Reminding Test, assessing episodic memory). In the presence of high amyloid loads, theta functional connectivity was decreased. It was suggested that amyloid deposits could negatively impact the extracellular environment, preventing compensatory adjustments. Moreover, Bajo et al. (2010) administered a memory task to healthy elderly MCI participants with similar performance levels than controls. They reported an increased interhemispheric gamma, beta, and alpha synchrony in MCI participants compared to controls. This increase in synchrony has been interpreted as reflecting compensatory adjustments in MCI individuals. The presence of interhemispheric activity is in line with the Hemispheric Asymmetry Reduction in Older (Cabeza et al., 2002) model. Finally, an overall increase in theta synchrony has been reported in fronto-occipital and right parietotemporal areas for MCI individuals during the performance of a mental arithmetic task, thus allowing the maintenance of cognitive performance (López et al., 2014a).

These resilience phenomena have been reported in patients with MCI, but may not always be necessary. Indeed, López et al. (2014b) showed that increases in synchrony were reduced in individuals with greater reserve.

### Resistance

Resistance refers to the prevention of brain injury and damage before their occurrence (Pascual-Leone and Bartres-Faz, 2021). AD is characterized by advanced brain and cognitive impairments, being the results of years of neurodegenerative processes, and therefore resistance phenomenon can no longer be observed. However, investigating the earlier stages of this disease, López et al. (2014a) used a mental arithmetic task (mental subtraction) at two levels of difficulty (subtracting from one to one, being the easy level, and subtracting from three to three being the hard level). Patients with MCI and controls both showed similar cognitive performance. Furthermore, lower synchrony at rest and increased delta synchrony in frontal interhemispheric regions during task completion (measured with PLV) were similar across groups. Thus, there seems to be maintenance of certain brain functions in early AD stages. However, such maintenance of specific brain communications was associated with compensatory increases in synchrony in other brain regions in patients with MCI. The concept of resistance, alone, would therefore not allow the maintenance of cognitive performance in individuals at risk of developing a neurodegenerative disease. Furthermore, increased synchrony in individuals with MCI was initially associated with preservation of brain and cognitive functioning, but may also be a sign of disease progression (Knyazeva et al., 2013).

### Maladaptive Changes

Despite the presence of maintained synchrony patterns and the ability of some individuals to compensate for disease-related brain damage, individuals with MCI also exhibit brain changes associated with impaired cognitive performance. For example, Caravaglios et al. (2018) showed that increases in beta synchrony in patients with MCI correlated with poorer performance on various neuropsychological tests (e.g., Trail Making Test, Rey Auditory Verbal Learning Test, and Semantic Verbal Fluency). Maladaptive changes thus occur and could be due to disturbances in the integrity of white matter fibers. Altered white matter integrity has indeed been shown to result in decreased alpha synchrony in individuals with MCI, particularly in parietal regions (Teipel et al., 2009). Synchrony analyses have also revealed decreased connectivity in frontal and parietotemporal alpha and beta activity, especially in interhemispheric couplings, associated with impaired cognitive performance (digit verbal span task; Li et al., 2019).

Thus, maladaptive changes appear to differ depending on AD progression level. Garn et al. (2015) revealed that qEEG markers such as decreased delta synchrony in parietal regions could have a predictive role in AD severity. Indeed, synchrony measures could discriminate between individuals with subjective cognitive decline (SCD; defined by a subjective complaint without cognitive impairment), MCI, and AD (Houmani et al., 2018). Using measures of synchrony (bump model) and complexity (epoch-based entropy), the authors discriminated patients with AD from patients with SCD and high specificity (91.6% accuracy, 100% specificity, and 87.8% sensitivity). Their results showed that patients with AD showed increased EEG synchrony in the theta band in frontal and occipital regions compared to patients with SCD. These differences could be explained by the degradation or lower cognitive reserve in patients with AD (Montemurro et al., 2021).

## Discussion

Results reviewed here highlight the importance of considering the temporal dynamics of brain activity, and synchronized brain communications, to our understanding of cognitive reserve, resilience, and maintenance in healthy and pathological aging. Dynamic connectivity patterns must display both stability and flexibility as a function of the cognitive process involved and task context. While the stability of dynamics in the network engaged is important to process relevant information, flexibility is required to adjust to goal changes and to inhibit the processing of irrelevant information (e.g., Voytek and Knight, 2015). Cognitive decline during healthy and pathological aging can result from impairments of such dynamic network activity. While further investigations remain necessary, the present review summarizes current work on the association of sustained, increased, or altered neural synchrony across brain regions, with cognitive preservation or decline.

While reserve has been associated with increased frontal synchrony in the theta and alpha bands (Ariza et al., 2015; Hong et al., 2016), resilience has been associated with increased alpha, beta, and gamma synchrony (e.g., Bajo et al., 2010). It is important to note that not all changes are associated with a relative preservation of cognitive performance. Indeed, maladaptive changes have also been reported, such as the increased delta synchrony in posterior regions (Jauny et al., 2022). Findings suggest that (1) the neural mechanisms involved in both reserve and resilience could be partly similar, and (2) resilience could involve a stronger involvement of higher frequency bands relative to reserve. This difference could be determined by the degree of preservation of the structural network integrity, which is modulated by the pathology progression, leading to different compensation levels. Yet, whether differences across reserve and resilience are mainly of quantitative nature, or also qualitative, remains to be further clarified.

Maintenance-related patterns were also observed in healthy older individuals, with a relative absence of impairments in frontoparietal communication dynamics associated with preserved cognitive performance (Rondina et al., 2019; Hinault et al., 2020, 2021). The brain's structure-function interplay appears to be at the heart of the heterogeneity associated with maintenance during aging (Courtney and Hinault, 2021; Jauny et al., 2022). Indeed, maintenance was observed with preserved dynamics in the relative absence of integrity loss. Conversely, reserve-related adjustments are a necessity in the presence of reduced structural integrity. The inability of any of these two mechanisms will lead to cognitive decline. Additional longitudinal studies remain necessary to further specify the concepts of maintenance and resistance mechanisms. Another open question for future research lies in the distinction between hyperactivation or hyperconnectivity resulting from (1) failed compensatory attempts, although some degree of network reorganization has been implemented, and (2) excitotoxic processes associated with the progression of neurodegenerative disorders. Direct investigations, associated with longitudinal follow-up, will be necessary to further clarify this distinction.

While these findings are promising, they only reflect a fraction of M/EEG aging studies. Future studies in the field could integrate the following recommendations to further address the contribution of brain dynamics. First, while resting-state measurements are indicators of the potential for brain networks' reorganization in cognitively challenging situations, they cannot always inform on their actual implementation and association with specific cognitive processes. Although few studies were conducted to address these issues, we expect that dynamic stability and flexibility of task-related networks could provide critical elements regarding subsequent cognitive changes and the effect of cognitive stimulation programs. In line with recent questioning in the field (e.g., Finn and Bandettini, 2021), it also appears critical to question whether resting-state recording can precisely measure elements of cognitive reserve. Although changes can be observed in brain activity at rest, specific task-related delays in the implementation, maintenance, or interruption of communications across brain regions could be highly sensitive to age and pathology effects. Recent work (Babiloni et al., 2020; Güntekin et al., 2021) highlighted the clinical value of investigating oddball-related brain activity to detect pathology effects. Finally, from an epidemiological perspective, future studies should also consider middle-aged individuals (40–60 years) to clarify the association between changes in brain dynamics and later cognitive trajectories. Indeed, age-related brain changes occur throughout life and changes in older individuals (>65 years) may be the result of earlier brain changes. Also, it appears critical that future studies include a more diverse population, especially in terms of education level (de Oliveira et al., 2018; Ashby-Mitchell et al., 2020). Indeed, most of the reviewed studies considered populations with a high level of education, which limits the investigation of reserve and resilience mechanisms.

Many studies relied on methods that are blind to temporal changes, such as amplitude comparison following averaging of large time periods. This facilitates data analyses but could lead to missing important information. Hidden-Markov models (Tibon et al., 2021) or multiscale entropy (McIntosh et al., 2014) enable the specification of time-varying connectivity states, which could further our understanding of the heterogeneity of cognitive aging. Finally, the majority of the reviewed studies involved sensor-based analyses and brain connectivity methods, such as coherence analyses. While the reviewed work reveals important patterns associated with resilience and maintenance, they are prone to volume conduction effects (e.g., Brunner et al., 2016) and suffer from low spatial accuracy. A more generalized use of non-biased connectivity estimates (e.g., Allouch et al., 2022), associated with source reconstruction of M/EEG activity, could clarify some open questions and replicate the currently reported findings. This would also enable the investigation of the same networks and regions of interest as previous fMRI work (Pascual-Leone and Bartres-Faz, 2021) to specify their time course and dynamics.

Brain connectivity changes observed in M/EEG could differentiate individuals according to pathology stages (Houmani et al., 2018) in a non-invasive manner. These data could also help predict pathology progression. Specifying rhythmic brain communication changes with age and pathology could also guide direct modulations of brain rhythms through targeted stimulations. Recent work, such as Reinhart and Nguyen (2019), revealed the feasibility of short-term interventions using transcranial alternative current stimulation (application of a low-intensity current to the brain with electrodes to synchronize or desynchronize brain oscillations) on working memory performance in healthy older adults. Improved working memory performance (up to 50 min post-stimulation) has been reported in healthy older adults following frontotemporal theta stimulation relative to a sham condition. Several points, such as the long-term benefits of these interventions, together with the optimal number of sessions, remain to be specified. Such knowledge could lead to a new specific and neurophysiologically grounded intervention targeting disrupted brain communications and enhancing rhythms associated with successful compensation, suggesting an intense development of this research field in the coming years.

## Author Contributions

GJ: investigation and writing. FE: supervision and review. TH: conceptualization, supervision, writing, and review. All authors contributed to the article and approved the submitted version.

## Conflict of Interest

The authors declare that the research was conducted in the absence of any commercial or financial relationships that could be construed as a potential conflict of interest.

## Publisher's Note

All claims expressed in this article are solely those of the authors and do not necessarily represent those of their affiliated organizations, or those of the publisher, the editors and the reviewers. Any product that may be evaluated in this article, or claim that may be made by its manufacturer, is not guaranteed or endorsed by the publisher.
